# Vesicle‐Associated Actin Assembly by Formins Promotes TGF*β*‐Induced ANGPTL4 Trafficking, Secretion and Cell Invasion

**DOI:** 10.1002/advs.202204896

**Published:** 2023-01-24

**Authors:** Dennis Frank, Christel Jessica Moussi, Svenja Ulferts, Lina Lorenzen, Carsten Schwan, Robert Grosse

**Affiliations:** ^1^ Institute of Experimental and Clinical Pharmacology and Toxicology Medical Faculty University of Freiburg 79104 Freiburg Germany; ^2^ Deutsche Forschungsgemeinschaft Research Training Group Membrane Plasticity in Tissue Development and Remodeling University of Marburg 35037 Marburg Germany; ^3^ Centre for Integrative Biological Signalling Studies – CIBSS 79104 Freiburg Germany

**Keywords:** actin dynamics, formins, invasion, TGF*β*−signaling, vesicle trafficking

## Abstract

Vesicle trafficking has emerged as an important process driving tumor progression through various mechanisms. Transforming growth factor beta (TGF*β*)‐mediated secretion of Angiopoietin‐like 4 (ANGPTL4) is important for cancer development. Here, Formin‐like 2 (FMNL2) is identified to be necessary for ANGPTL4 trafficking and secretion in response to TGF*β*. Protein kinase C (PKC)‐dependent phosphorylation of FMNL2 downstream of TGF*β* stimulation is required for cancer cell invasion as well as ANGPTL4 vesicle trafficking and secretion. Moreover, using super resolution microscopy, ANGPTL4 trafficking is actin‐dependent with FMNL2 directly polymerizing actin at ANGPTL4‐containing vesicles, which are associated with Rab8a and myosin Vb. This work uncovers a formin‐controlled mechanism that transiently polymerizes actin directly at intracellular vesicles to facilitate their mobility. This mechanism may be important for the regulation of cancer cell metastasis and tumor progression.

## Graphical abstract

In this study, a novel role for the formin FMNL2 is described. Secretion of oncogenic factors into the tumor microenvironment is an essential process for cancer development. TGF*β*‐induced invasion and the secretion of the pro‐metastatic factor ANGPTL4 rely on phosphorylation of FMNL2 by PKC*α*. Structured illumination microscopy (SIM) reveals FMNL2‐dependent actin dynamics at ANGPTL4‐containing vesicles for efficient intracellular trafficking and subsequent secretion.

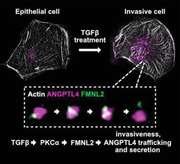

## 1 Introduction

The dynamic regulation of the actin cytoskeleton is of utmost importance for cells ability to migrate. While cellular movement is necessary for essential steps during embryogenesis and wound healing, it is also an important characteristic of tumor cells, that leads to the development of metastatic cancers. While secretory proteins have been described as essential components of the tumor microenvironment, not much is known about the involvement of actin in the traffic and secretion of factors promoting cancer cell progression.

Formins are a major group of actin regulators and are classified by the presence of a highly conserved formin homology domain 2 (FH2), which is crucial for their ability to nucleate and elongate unbranched actin filaments.^[^ ^1^, ^2^ ^]^ Formin‐like 2 (FMNL2) belongs to the diaphanous‐related formins (DRFs), a sub‐group of the formin family that is regulated by autoinhibition. This autoinhibited state is facilitated through intramolecular interactions between the C‐ and N‐terminus.^[^ ^3^ ^]^ Binding of Rho‐GTPases to the GTPase binding domain, as well as post‐translational modifications, such as phosphorylations at diverse sites, can release the autoinhibited state and modulate the activity of FMNL2.^[^ ^4^, ^5^, ^6^, ^7^ ^]^

Co‐translational N‐myristoylation is indispensable for FMNL2 to localize at the plasma membrane, where it can regulate actin dynamics and contribute to morphological changes of the cell.^[^ ^8^ ^]^ FMNL2 localizes largely at protrusive structures, such as filopodia and lamellipodia.^[^ ^9^ ^]^ Besides their well‐described involvement in cell migration and tumor invasion, these structures are also needed for the formation of cell‐cell contact sites in epithelial cells.^[^ ^10^, ^11^ ^]^ Together with its family member FMNL3, FMNL2 was shown to maintain the structural integrity of the Golgi apparatus and anterograde trafficking of transport vesicles.^[^ ^12^ ^]^ In context of intracellular trafficking, the actin cytoskeleton together with formins has been mostly described to be involved in endocytotic processes and endosomal dynamics, although transient actin dynamics were shown to provide mechanical forces for the scission of transport carriers from the trans‐Golgi network, indicating a role of actin dynamics for the secretory pathway.^[^ ^13^, ^14^, ^15^ ^]^ FMNL2 has been implicated in several cancers, for example, in colorectal cancer and nasopharyngeal carcinoma FMNL2 upregulation was shown to promote invasiveness. Silencing FMNL2 in gastric cancer and melanoma suppressed cancer cell migration.^[^ ^16^, ^17^, ^18^, ^19^ ^]^ Previous work revealed that downstream of Rac1 activity, FMNL2 binds to components of the adherence junction complex at newly forming cell‐cell contact sites, where it drives the assembly of junctional actin.^[^ ^4^ ^]^ In contrast to this pro‐epithelialization function of FMNL2, we also showed that PKC*α*‐dependent phosphorylation at a specific C‐terminal serine residue promotes trafficking of integrins, a necessary process in cancer cell invasion.^[^ ^7^ ^]^

In this study, we uncover a TGF*β*‐induced activation of FMNL2 via its phosphorylation by PKC*α* and/or PKC*β* and show that FMNL2 is necessary for TGF*β*‐induced cancer cell invasion into 3D matrices and the secretion of the pro‐metastatic factor Angiopoietin‐like 4 (ANGPTL4).^[^ ^20^, ^21^ ^]^ Structured illumination microscopy (SIM) in living cells revealed that FMNL2 directly polymerizes actin on mobile ANGPTL4‐containing vesicles in a highly dynamic manner for their proper intracellular trafficking and subsequent secretion. Conclusively, we report a novel role of vesicular actin dynamics that may contribute to the progression of cancers.

## 2 Results and Discussion

**Figure 1:**
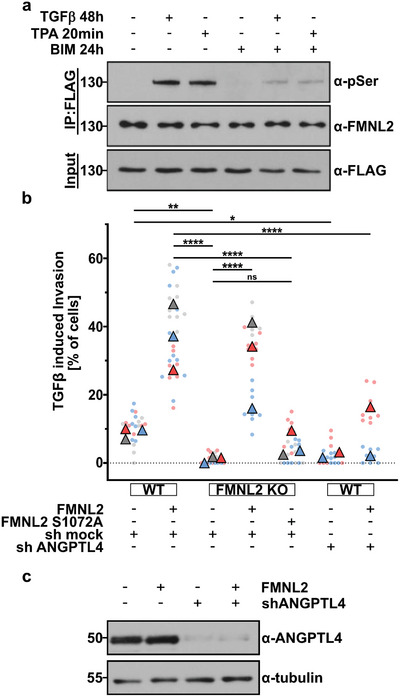
TGF*β*‐induced phosphorylation of FMNL2 at S1072 by PKC*α* is required for invasion. a) Immunoprecipitation of FMNL2‐FLAG with an anti‐FLAG antibody from lysates of stably FMNL2‐FLAG‐expressing MCF10A cells after stimulation with 4 ng mL^−1^ TGF*β* or 200 nm TPA, or inhibitory treatment with 2 µm BIM. Phosphorylated FMNL2 was detected by western blot with a phospho‐(Ser)‐specific antibody. In the expression control, an anti‐Flag antibody was used and in the IP control an anti‐FMNL2 antibody was used. b) Inverted transwell invasion assay with MCF10A cells after 48 h TGF*β* stimulation. As indicated WT or FMNL2 KO cells were transfected with FMNL2, FMNL2 (S1072A), a shRNA targeting ANGPTL4 or a non‐targeting shRNA. Percentage of invaded cells in relation to the total cell number was quantified. Graph displays technical replicates (*n* ≥ 5) as dots (each color represents replicates from different experimental days shown as triangles). One‐way ANOVA with Tukey's multiple comparisons test was used for statistical analysis (^*^= *p* < 0.05). c) WT cells without transfection or expressing FMNL2 were analyzed for ANGPTL4 expression after shRNA transfection targeting ANGPTL4 by Western blot.

To assess the potential role for FMNL2 in TGF*β* signaling, we analyzed its serine phosphorylation after TGF*β* stimulation. Immunoprecipitation of FMNL2‐FLAG from stably expressing MCF10A cells (Figure S1a, Supporting Information) showed a prominent increase in phosphorylation after TGF*β* addition, which could be blocked by the PKC inhibitor BIM (**Figure** **1**a). TPA stimulation served as a positive control. Thus, FMNL2 is a target of TGF*β* signaling via protein kinase C. In our previous studies, only silencing of PKC*α* resulted in a significant reduction of FMNL2 serine‐phosphorylation after TPA‐treatment. Moreover, in the FMNL2‐S1072A mutant no other serine residues were phosphorylated in response to PKC activation by TPA. These previous results indicate that PKC*α* is the dominant isozyme that specifically phosphorylates FMNL2 in cells.^[^ ^7^ ^]^ In MCF10A cells increasing durations of TGF*β* stimulation showed no changes in the expression levels of PKC*α* (Figure S1b, Supporting Information). We next performed 3D Matrigel invasion assays to address the functionality of this signaling axis. For this, we used FMNL2 knock‐out cells generated by CRISPR/Cas‐mediated gene deletion.^[^ ^22^ ^]^ Stimulation of cells with TGF*β* resulted in an increase of invasion when FMNL2 was stably overexpressed whereas invasive migration was abrogated in FMNL2 KO cells (Figure 1b). This phenotype could be rescued by re‐expressing FMNL2 but not by a FMNL2‐S1072A lacking the PKC phosphorylation site. In these experiments, both FMNL2 constructs were stably re‐expressed at similar levels in the FMNL2 KO cell line (Figure S1c, Supporting Information). Control invasion assays revealed that MCF10A cells lack invasive capabilities in the absence of TGF*β* (Figure S1d,e, Supporting Information). These data show that FMNL2 phosphorylation is essential for TGF*β*‐induced cell motility in transformed MCF10A breast epithelial cells.

**Figure 2:**
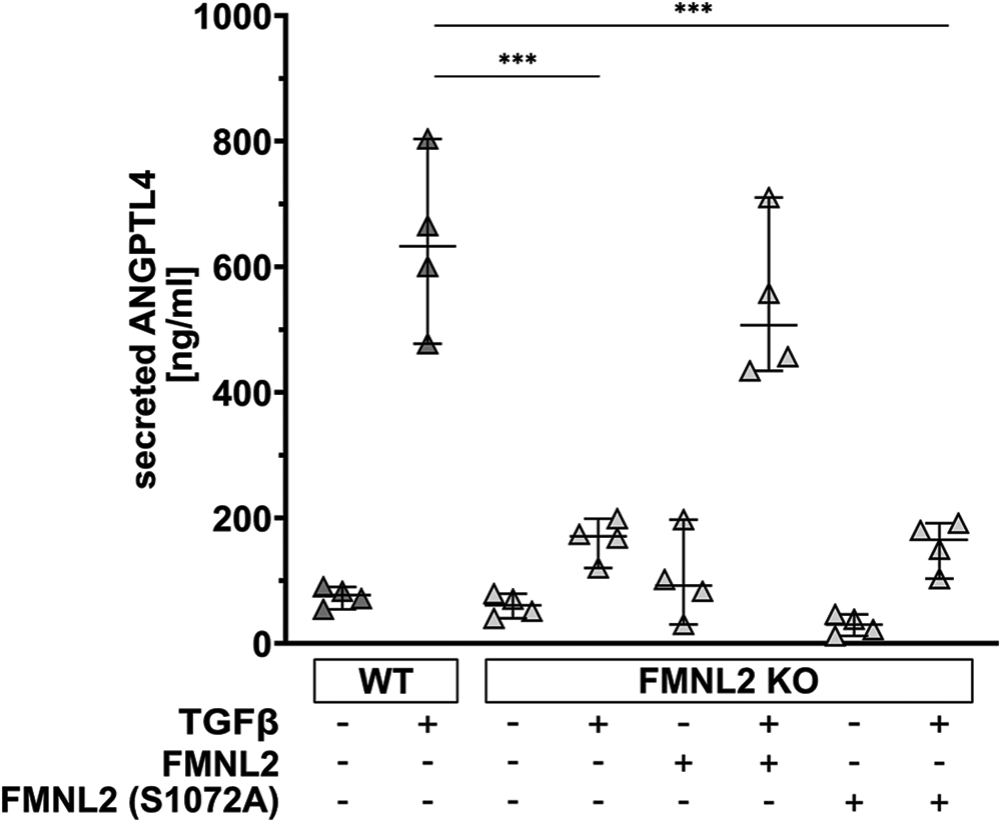
TGF*β*‐induced secretion of ANGPTL4 requires phosphorylation of FMNL2 at S1072 by PKC*α*. The secretion of ANGPTL4 by MCF10A wildtype‐, FMNL2 knockout‐, and FMNL2 rescue cells was analyzed by ELISA assay. Cells were treated with TGF*β* for 48 h. The mean (black line) concentrations of secreted ANGPTL4 in the supernatant is shown for 4 independent biological replicates (black line, *n* = 4; ±SEM) as well as the mean of individual replicates (triangles). One‐way ANOVA with Tukey's multiple comparisons test was used for statistical analysis (^***^= *p* < 0001).

TGF*β* can promote tumor spread via ANGPTL4.^[^ ^21^ ^]^ ANGPTL4 is a secreted glycoprotein overexpressed in many tumors that promotes an invasive and metastatic phenotype.^[^ ^23^, ^24^, ^25^ ^]^ We therefore measured TGF*β*‐induced ANGPTL4 secretion in the absence or presence of FMNL2. Interestingly, ANGPTL4 secretion was significantly inhibited in FMNL2 KO cells, which could be rescued by re‐introducing FMNL2 but not FMNL2‐S1072A (**Figure** **2**). As expected, knock‐down of ANPTL4 resulted in decreased TGF*β*‐induced invasive capacity (Figure 1b). This clearly demonstrates that TGF*β*‐induced ANGPTL4 secretion, similarly to invasive migration, depends on FMNL2 and its PKC phosphorylation site and thus argues for a functional link between FMNL2‐guided tumor cell motility and TGF*β*‐mediated ANGPTL4 secretion.

**Figure 3:**
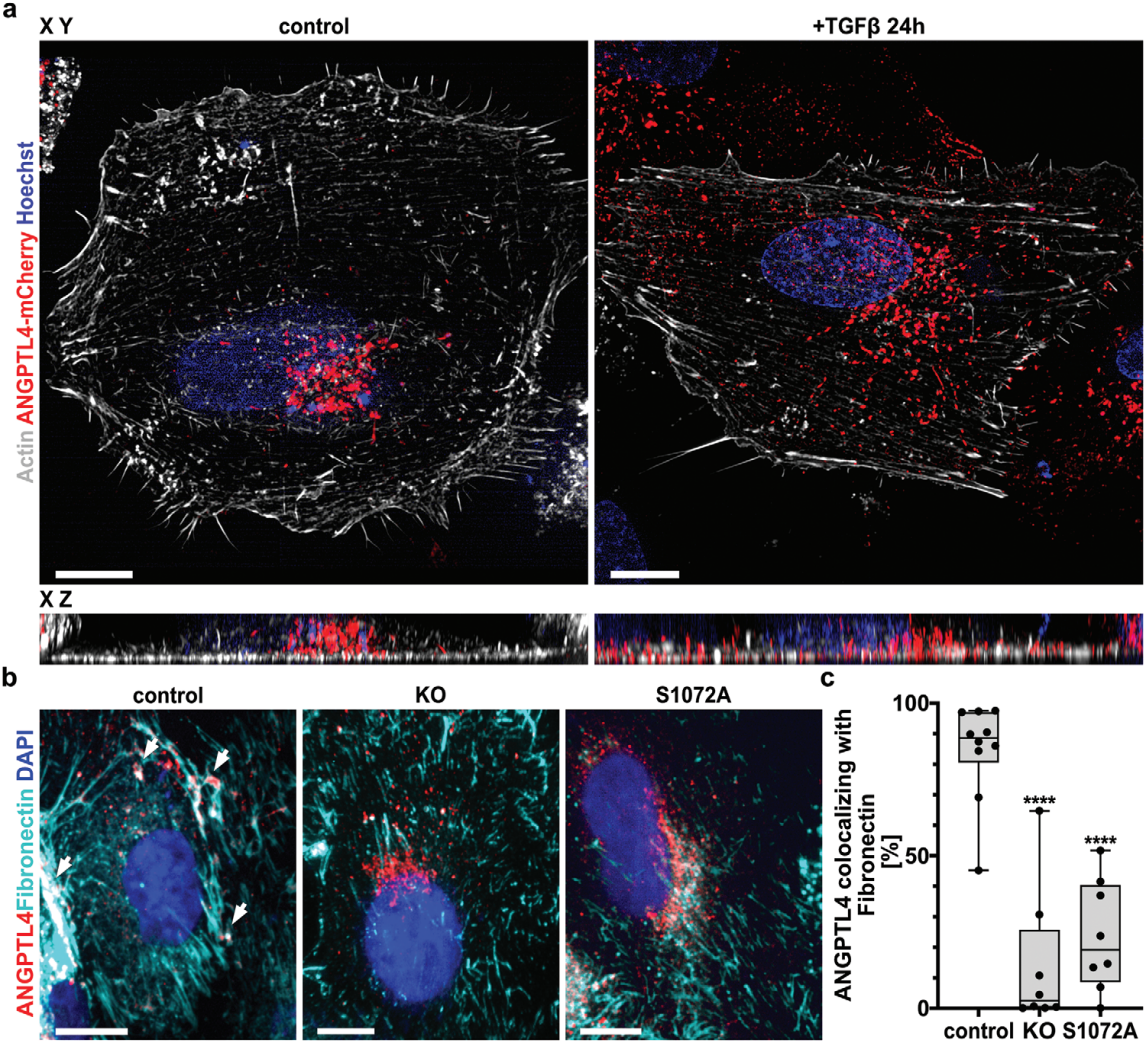
TGF*β*‐induced trafficking of ANGPTL4 requires phosphorylation of FMNL2 at S1072. a) MCF10A wildtype cells were transfected with ANGPTL4‐mCherry and actin‐chromobody SNAP. Maximum intensity projections (MIP) of SIM images show overview (XY) and sideview (XZ) of ANGPTL4‐mCherry distribution after 24 h in untreated control cells (left panel) and cells stimulated with TGF*β* for 24 h (right panel). b) Immunostainings of fibronectin and ANGPTL4 in MCF10A cells (control = wildtype; KO = FMNL2‐KO; S1072A = FMNL‐ KO + FMNL2 S1072A) after 48 h stimulation with TGF*β*. White arrows in control cells (left panel) indicate colocalization of secreted ANGPTL4 and fibronectin. Scale bar = 10 µm. c) Quantification of ANGPTL4 colocalizing with fibronectin. The relative area of ANGPTL4 that is also positive for fibronectin was quantified. Cell lines from (b) were analyzed: Fields of view for control (*n* = 10), KO (*n* = 8), and S1072A (*n* = 8) from 2 independent stainings. Data shown as box plot (median, minimum to maximum). One‐way ANOVA with Tukey's multiple comparisons test was used for statistical analysis (^****^= *p* < 0.0001).

To investigate ANGPTL4 trafficking more directly, we expressed ANGPTL4‐mCherry and visualized its intracellular distribution. Notably, stimulation with TGF*β* not only caused an apparent spreading of ANGPTL4 vesicles throughout the cytoplasm but also increasing amounts of ANGPTL4 were found outside the cell (**Figure** **3**a). We therefore stained for the extracellular matrix protein fibronectin and found that ANGPTL4 accumulated at sites of strong fibronectin depositions (Figure 3b,c). In contrast, ANGPTL4 vesicles did not distribute throughout the cells and secreted ANGPTL4 did not associate with the fibronectin matrix in FMNL2 KO cells or FMNL2‐S1072A cells (Figure 3b,c), indicating that FMNL2 was involved in ANGPTL4 vesicle trafficking downstream of TGF*β*.

**Figure 4:**
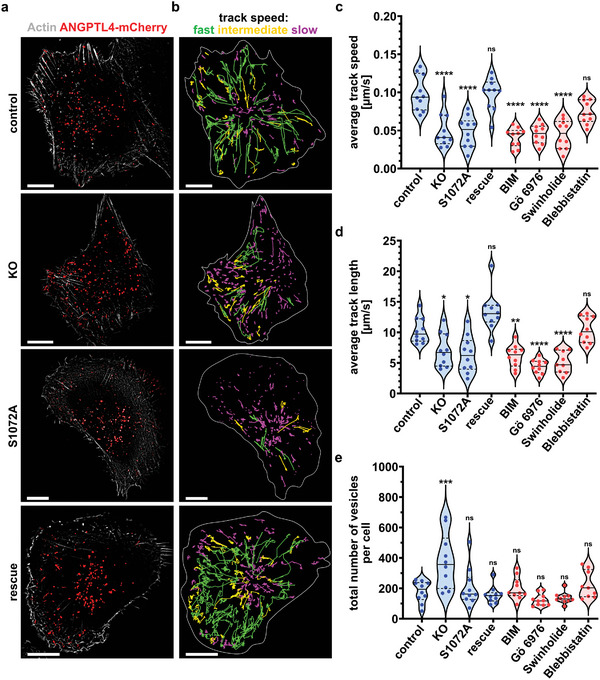
Super‐resolution live cell imaging and tracking of ANGPTL4‐mCherry‐containing vesicles in MCF10A cells after TGF*β* stimulation. a) MCF10A cells (Control = wildtype; KO = FMNL2‐KO; S1072A = FMNL2‐KO +FMNL2S1072A‐GFP; Rescue = FMNL2‐KO +FMNL2‐GFP) were transfected with ANGPTL4‐mCherry and actin‐chromobody SNAP. Cells were stimulated for 24 h with TGF*β* (4 ng mL^−1^). These cells were subjected to super‐resolution live cell imaging with enhanced temporal resolution (burst mode, sliding processing). b) In the resulting sequential images, ANGPTL4‐positive vesicles were tracked. The trajectories of ANGPTL4‐mCherry vesicles tracked for at least 60 s are shown. Trajectories were categorized by their average track speed into three groups: magenta = slow speed (0–0.10 µm s^−1^), yellow = intermediate speed (0.10 – < 0.15 µm s^−1^), green = fast speed (≥0.15 µm s^−1^). Grey line indicates cell border based on the actin cytoskeleton. Scale Bar = 10 µm. c–e) In cells treated as in (a), the average track speed (c), average track length (d), and number of vesicles (e) was quantified. The following cell lines were used (blue): Control = MCF10A WT (*n* = 10), KO = MCF10A FMNL2‐KO (*n* = 10), S1072A = MCF10A FMNL2‐KO (+FMNL2S1072A‐GFP) (*n* = 10) and rescue = MCF10A FMNL2‐KO (+FMNL2‐GFP) (*n* = 10). WT cells were additionally treated with (red): PKC inhibition (BIM, 2 µm, 30 min, *n* = 10 and Gö6976, 1 µm, 60 min, *n* = 10), disruption of the actin cytoskeleton (Swinholide, 30 nm, 30 min, *n* = 9) and inhibition of Myosin II (Blebbistatin, 30 µm, 30 min, *n* = 10). Data are shown as violin plots with median (solid line), first and second quartile (dashed lines), spots represent individual values. One‐way ANOVA with Tukey's multiple comparisons test was used for statistical analysis (^*^= *p* < 0.05; ^**^= *p* < 0.01; ^***^= *p* < 0.001; ^****^= *p* < 0.0001).

Next, we aimed to analyze ANGPTL4 trafficking directly in living cells by tracking individual ANGPTL4‐containing vesicles after TGF*β* treatment in control or FMNL2 KO cells and cells re‐expressing either FMNL2‐GFP or FMNL2‐S1072A‐GFP. For this, we used structured‐illumination‐microscopy (SIM) that allows for high spatiotemporal resolution. Using this approach, we found that ANGPTL4 trafficking depends on FMNL2 (**Figure** **4**a,b; Videos S1 and S2, Supporting Information). Interestingly, FMNL2 determined the average vesicle speed as well as track length of ANGPTL4 trafficking routes in response to TGF*β* (Figure 4b–d). Moreover, this was sensitive to PKC inhibition or depolymerization of actin filaments using the BIM or Swinholide, respectively, while inhibition of myosin II using Blebbistatin had no effect. Treatment with the ATP‐competitive PKC inhibitor Gö6976, specific for PKC isozymes *α* and *β*1, resulted in the same trafficking defect (Figure 4b–d). Notably, similar trafficking effects as well as FMNL2 phosphorylation were already observed 1 h after stimulation with TGF*β* indicative for a more direct regulatory mechanism (Figure S2, Supporting Information). These data show that intracellular trafficking of ANGPTL4 vesicles requires formin‐dependent actin polymerization as well as PKC activity.

Next, the number of ANGPTL4‐positive vesicles per tracked cell were counted, revealing an increase of vesicles in cells lacking FMNL2 (Figure 4e). The number of vesicles returned to wildtype levels after reintroducing FMNL2 into the knockout cell line. This phenotype suggests that the loss of FMNL2 leads to the impaired intracellular trafficking and subsequently to the failed secretion of ANGPTL4, resulting in a vesicle accumulation inside the cell. As expected, the number of ANGPTL4‐containing vesicles did not change for cells treated for 30 min with either Swinholide, Blebbistatin, BIM or 60 min Gö6976 due to functioning intracellular trafficking of ANGPTL4 up until drug treatment (Figure 4e). Surprisingly, the number of intracellular vesicles was also reduced after reexpression of FMNL2‐S1072A. We can only speculate that additional phosphorylation independent effects at the golgi might reduce the formation of ANGPTL4‐containing vesicles resulting in less accumulation.^[^ ^12^ ^]^ Additionally, altered transcriptional and/or translational regulation of ANGPTL4 may account for this phenomenon.

After finding that the TGF*β*‐induced secretion and vesicular trafficking of ANGPTL4 depends on FMNL2, we wanted to further investigate the trafficking route, to which ANGPTL4 is assigned to. Characterizing the secretion pathway of ANGPTL4 could provide a better understanding on FMNL2s’ involvement in vesicular trafficking.

**Figure 5:**
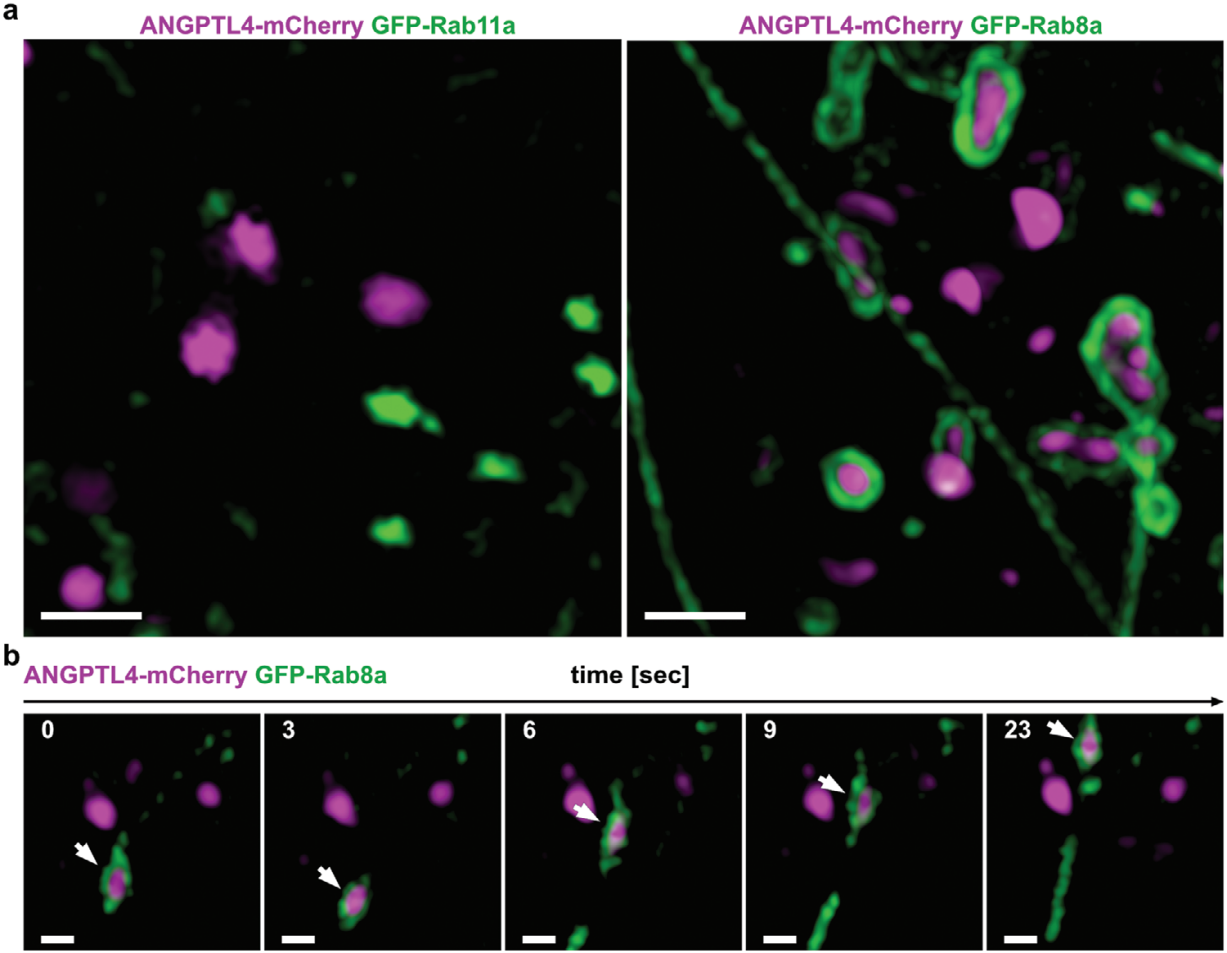
GFP‐Rab8a colocalizes with ANGPTL4‐mCherry during vesicle transport. a) Sections of MCF10A wildtype cells transfected with ANGPTL4‐mCherry and either GFP‐Rab11 (left panel) or GFP‐Rab8a (right panel). SIM images show colocalization of ANGPTL4‐mCherry with GFP‐Rab8a, but not with GFP‐Rab11. Scale bar = 1 µm. b) Time sequence shows co‐trafficking of an ANGPTL4‐mCherry (magenta) containing vesicle with Rab8a‐GFP (green; white arrow) in MCF10A wildtype cells. Scale bar = 200 nm.

The Rab family of Ras‐related GTPases plays a key role in the regulation of intracellular trafficking. Dysregulations of Rab GTPases have been associated with tumor progression.^[^ ^26^ ^]^ Rab11a is used as a marker for exosomes as well as recycling endosomes, while Rab8a has been shown to regulate a secretion pathway from the trans‐Golgi network to the plasma membrane as well as endosomal recycling pathway.^[^ ^27^, ^28^, ^29^ ^]^ We hence performed co‐expression experiments with either GFP‐Rab11a or GFP‐Rab8a together with ANGPTL4‐mCherry and observed, that ANGPTL4 was localized to a GFP‐Rab8a‐positive vesicle compartment, whereas Rab11a vesicles showed no colocalization with ANGPTL4 indicating that ANGPTL4s’ secretion pathway acts independent of Rab11a (**Figure** **5**a). Stainings for endogenous Rab8a and ANGPTL4 revealed similar localizations (Figure S4, Supporting Information). In FMNL2‐KO cells, the colocalization of Rab8a with ANGPTL4 vesicles was not disturbed (Figure S3, Supporting Information). However, the vesicles had a decreased motility as mentioned above (Figure 4a–d). In WT cells, we observed mobile vesicles co‐trafficking ANGPTL4 and Rab8a (Figure 5b). It is tempting to assume that FMNL2 is involved in the regulation of Rab8a‐dependent trafficking routes, such as the exocytic pathway of newly synthesized matrix metalloproteinase‐14 (MMP‐14), a well‐characterized factor contributing to cancer progression.^[^ ^30^ ^]^ In addition, MMP‐14s’ trafficking was shown to be regulated in a Rab8‐dependent manner for invasion into 3D matrices in both breast cancer cells and macrophages.^[^ ^31^, ^32^ ^]^

**Figure 6:**
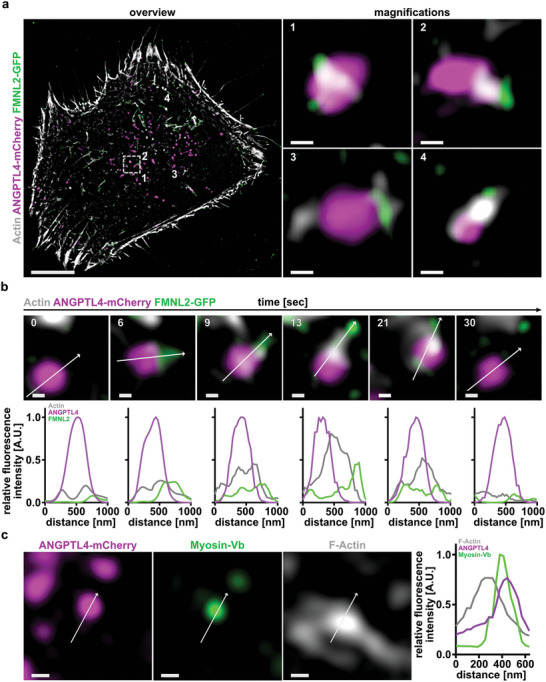
Visualization of actin at ANGPTL4‐positive vesicles. a) MCF10A rescue cells (FMNL2 KO + FMNL2‐GFP, green) were transiently transfected with ANGPTL4‐mCherry (magenta) and Actin‐Chromobody SNAP (white) and stimulated for 24 h with TGF*β*. Cells were subjected to super‐resolution live cell imaging with enhanced temporal resolution. White numbers (1, 2, 3, and 4) in overview image (left panel) indicate location of magnified ANGPTL4‐mCherry containing vesicles colocalizing with actin and FMNL2‐GFP. Scale bar = 10 µm, magnification = 200 nm. b) Time sequence of a vesicle from (a) (white box) displaying actin polymerization (white) by FMNL2 (green) at ANGPTL4‐mCherry‐containing vesicle (magenta). For each timepoint, corresponding line‐scan profile was added below. White arrows indicate line scan direction and length. Line‐scan profiles show normalized fluorescence intensity of actin (grey), ANGPTL4 (magenta), and FMNL2 (green). c) Immunostaining of Myosin‐Vb (green) in MCF10A wildtype cells that were treated with TGF*β* for 24 h after transfection with ANGPTL4‐mCherry (magenta). Phalloidin was used to visualize F‐actin (white). White arrows indicate line scan direction and length. Line‐scan profile shows normalized fluorescence intensity of ANGPTL4 (magenta), Myosin‐Vb (green), and F‐Actin (grey). Scale bar = 200 nm.

Since ANGPTL4 vesicle trafficking was formin and actin dependent we wanted to directly evidence this process in living cells. For this, we co‐expressed ANGPTL4‐mCherry and FMNL2‐GFP as well as an anti actin‐nanobody, termed actin‐Chromobody, to detect endogenous actin dynamics. TGF*β*‐stimulated cells were imaged using SIM at a temporal resolution of 19 frames per second over a period of 3 min. We readily detected ANGPTL4 vesicles that associated with F‐actin in a highly dynamic manner (Video S3, Supporting Information). Actin filaments were clearly attached to FMNL2 molecules (**Figure** **6**a). Notably, FMNL2‐mediated actin assembly at ANGPTL4 vesicles was very transient over time periods of a few seconds before FMNL2 dissociated and actin could no longer be detected (Figure 6b). This demonstrates that formin‐mediated actin assembly at these vesicles is a very dynamic and transient process rather than a continuous transport of the vesicle along prominent actin fibres over expanded time periods.

Unconventional myosin motors, like class V myosins, play critical roles in efficient trafficking of a vareity of intracellular cargoes.^[^ ^33^ ^]^ Moreover, Myosin‐Vb has been shown to interact with Rab8a and to be involved in several exocytotic events together with Rab8a.^[^ ^34^, ^35^, ^36^ ^]^ Myosin‐Vb dependent transport of vesicles has been previously observed in mouse oocytes.^[^ ^37^ ^]^ Hence, we aimed to investigate if ANGPTL4‐containing vesicles associate with the actin cytoskeleton through the Myosin‐Vb motor protein. Indeed we were able to detect colocalization of ANGPTL4‐containing vesicles with Myosin‐Vb at actin structures (Figure 6c). Similar to Rab8a, the recruitment of Myosin‐Vb to ANGPTL‐containing vesicles was not affected by the FMNL2 KO (Figure S5, Supporting Information). However, the vesicles had a reduced motility. Taken together, these results indicate that critical components for a secretory trafficking pathway including Rab8a and Myosin‐Vb are recruited to ANGPTL4‐positive vesicles, and FMNL2 is involved in regulating the intracellular trafficking by polymerizing actin on ANGPTL4‐containing vesicles.

We suggest that FMNL2 creates pushing forces at vesicles destined for secretion through dynamic and transient actin polymerization. Several types of motor proteins are bound to transport vesicles, which enable track switching from actin filaments to microtubules and vice versa.^[^ ^38^, ^39^ ^]^ These assistive forces created by FMNL2 could result in the dissociation of motor proteins with the current cytoskeletal element they are associated with, pushing the transport vesicle toward the correct secretory pathway. When cells lack FMNL2, the ANGPTL4‐containing vesicles cannot reach their proper destination and thus secretion fails.

## 3 Conclusion

We find that phosphorylated FMNL2 drives TGF*β*‐induced invasion of breast tumor cells by critically promoting secretion of the pro‐metastatic factor ANGPTL4. Phosphorylation of FMNL2 downstream of TGF*β*‐stimulation and PKC activation is a critical event to promote this pro‐invasive programme. The mechanism requires dynamic and transient actin assembly at secretory vesicles facilitated by FMNL2. In the absence of FMNL2 and/or localized actin, polymerization vesicles loose their motility thereby preventing ANGPTL4 secretion. ANGPTL4 follows a defined secretory pathway via a Rab8‐positive vesicle compartment. The Rab8‐interacting unconventional Myosin‐Vb is a highly processive motor that is recruited to ANGPTL4‐containing vesicles. As a consequence, active force can be generated at these vesicles which might be required for trafficking, track switching, or anchoring.

In summary, we report a novel role for FMNL2 in vesicular actin dynamics that contributes to a pro‐invasive programme and may promote the progression of cancers.

## 4 Experimental Section

### Cell Lines, DNA, Plasmids

MCF10A cells were cultured in DMEM/F12 (Gibco Life Technologies) supplemented with 5% horse serum, 20 ng mL^−1^ epidermal growth factor, 10 µg mL^−1^ insulin, 0.5 µg mL^−1^ hydrocortisone, 100 ng mL^−1^ cholera toxin, 100 U mL^−1^ penicillin, and 100 µg mL^−1^ streptomycin. HEKT293T cells were maintained in DMEM supplemented with 10% fetal bovine serum, 100 U mL^−1^ penicillin, and 100 µg mL^−1^ streptomycin.

MCF10A stable cell lines were generated by lentiviral transduction. Viruses were produced by transfecting HEKT293T cells with lipofectamine 2000 according to manufacturer's instructions with packaging plasmid psPAX2, envelope plasmid pMD2G and expression plasmids: pEGFP‐C1‐Rab11a (Choudhury et al., 2002),^[^ ^40^ ^]^ pGFP‐Rab8a (Nachury et al., 2007),^[^ ^41^ ^]^ PLVX‐FMNL2‐FLAG, PWPXL‐FMNL2‐GFP (Wang et al., 2015), PWPXL‐FMNL2‐S1072A‐GFP (Wang et al., 2015),^[^ ^7^ ^]^ and pTRIPZ shANGPTL4_4 RFP (Dharmacon). Supernatants were harvested 48 h after transfection and filtered through 0.45 µm filter. MCF10A cells were infected by the virus supernatant. Forty eight hours after infection, cells were trypsinized and passaged. They were either FACS‐sorted to maintain a homogeneously expressing population of cells or underwent selection with 2.5 µg mL^−1^ puromycin.

All constructs were cloned by restriction enzyme digest technique. ANGPTL4‐V5 (Addgene) was cloned into pmCherry‐N1 vector (Clonetech). Following primers were used for cloning of ANGPTL4‐mCherry: ANGPTL4 rev: gcggccgctcaggaggctgcctctgctgccat; ANGPTL4 fwd: gtcgactcagcggtgctccgacggccggg. FMNL2‐FLAG and FMNL2‐S1072A‐FLAG both were cloned from PWPXL FMNL2‐GFP or PWPXL FMNL2‐S1072A‐GFP (Wang et al.,2015) into a PLVX vector. Following primers were used for both constructs: FMNL2‐FLAG rev: ‐tctagattacttgtcgtcatcgtccttgtaatccatggagcccattgttatttcggcaccatt; FMNL2‐FLAG fwd: ctcgagcatgggcaacgcagggagcatgg.

### Western Blot

Proteins were separated by sodium dodecyl sulfate polyacrylamide gel electrophoresis (SDS‐PAGE). From 8% to 12% separating gels were used according to the different sizes of the proteins. Proteins from SDS‐PAGE were transferred into nitrate cellulose membranes. After the transfer, membranes were placed into blocking buffer and incubated for 1 h at room temperature. Different primary antibodies were diluted in the blocking buffer and incubated with the membranes on a shaker for 1–2 h at room temperature or overnight at 4 °C. Horseradish peroxidase‐labeled secondary antibody was added for 1 h at room temperature. Ultrasensitive enhanced chemiluminescence (ECL) substrate was used to detect the proteins. Primary antibodies used were anti‐ FLAG‐HRP (1:5000, mouse monoclonal, Sigma‐Aldrich), anti‐FMNL2 (1:250, mouse monoclonal, SCB), anti‐PKC*α* (1:1000, rabbit polyclonal, CST), anti‐PKC(S) (1:1000, rabbit monoclonal, CST), and anti‐Tubulin (1:1000, rabbit monoclonal, CST). Secondary antibody used was anti‐rabbit IgG‐HRP (1:5000, goat, Biorad).

### Immunoprecipitation

Cells were harvested for immunoprecipitation analysis with lysis buffer (20 mm Tris‐HCl (pH 7.4), 150 mm NaCL, 2 mm EDTA, 0.1% ASB‐14, complete protease and phosphatase inhibitors). After centrifugation (20 000 rpm, 15 min at 4 °C) supernatants were collected incubated with Flag‐conjugated agarose beads (Sigma) for 90 min at 4 °C. Immunoprecipitates were centrifuged and washed four times with lysis buffer. After 2x Laemmli buffer was added, samples were subjected to SDS‐PAGE and Western blotting analysis.

### Immunofluorescence

MCF10A cells were fixed in 4% formaldehyde for 20 min, permeabilized in 0.15% Triton X‐100/PBS for 10 min and blocked in blocking solution (1% BSA, 0.05% Tween 20, PBS) for 30 min at room temperature. Primary antibodies diluted in blocking solution were added for 1 h at room temperature and detected by fluorochrome‐conjugated secondary antibodies. Nuclei were stained DAPI (Sigma–Aldrich). Primary antibodies used were anti‐ANGPTL4 (1:1000, rabbit monoclonal, Sigma–Aldrich), anti‐Fibronectin (1:1000, mouse monoclonal, Sigma‐Aldrich), anti‐FLAG M2 (1:250, mouse monoclonal, Sigma–Aldrich) and anti‐Rab8 (1:100, mouse monoclonal, Biolegend). Secondary antibodies used were Alexa Fluor 488 chicken anti‐mouse IgG (H+L) (1:400, Thermo Fisher Scientific), Alexa Fluor 488 chicken anti‐rabbit IgG (H+L) (1:400, Thermo Fisher Scientific), and Alexa Fluor 568 donkey anti‐mouse IgG (H+L) (1:400, Thermo Fisher Scientific).

### Enzyme‐Linked Immunosorbent Assay (ELISA)

Human ANGPTL4 Duoset ELISA kit (BioRad) was used according to manufacturer's instructions to determine levels of secreted ANGPTL4 after different treatments. Absorbance was read at 450 nm in a plate reader.

### Inverted Transwell Invasion Assay

Inverted Transwell invasion assays were performed as described (Kitzing et al., 2010).^[^ ^6^ ^]^ Doxycycline was used to induce the expression of shRNAs prior to the invasion assay. Cells were seeded for 48 h before the assay and stimulated with TGF*β*. Upper chambers of the Transwell inserts were coated with 50 µL growth factor reduced Matrigel (BD Biosciences) and polymerized for 60 min at 37 °C. The inserts were inverted allowing cell seeding (10,000 cells per inserts for MCF10A cells) and adhering on the outer bottom. After 1 h, the Transwell inserts were reverted. The upper chambers were filled with medium containing or void of TGF*β* based on the condition studied. Cells were allowed to invade for 48 h before fixation, permeabilization and subsequent staining with DAPI and phalloidin 488. Confocal z‐stacks of 100 µm were acquired every 5 µm for nine random imaging fields of each insert with LSM 700 confocal microscope (Zeiss), using the 40X objective and the ZEN software (Zeiss). Cells were considered to be invasive when they invaded at least 15 µm into transwell. Nuclei of invading and non‐invading cells were counted in ImageJ with the “analyze particle” function.

### Microscopy, Live Imaging, and Image Analysis

MCF10A cells were seeded in 35 mm glass bottom dishes (Greiner) and were subjected for live imaging 24 h after TGF*β* treatment and transfection with ANGPTL4‐mCherry and Actin‐Chromobody SNAP plasmids using Lipofectamine 3000 according to manufacturer's instructions. SNAP‐Cell 647 SiR (1:2000, NEB, S9192S) was added, followed by 30 min incubation before imaging. Hoechst 33 342 was directly added (1:1000, Invitrogen, R37605) into the dishes for staining of the nuclei. Structured illumination microscopy (SIM) imaging was performed with an ELYRA 7 microscope (Zeiss) equipped with a 63  ×  1.4 Oil DIC objective and a Pecon incubation chamber, providing a stable environment for the samples at 37 °C and 5% CO2. All acquired imaging data were SIM processed using Zen 3.0 black edition and analyzed with Imaris 9.8.0.

### Tracking

Tracking was performed with the spot tracking function of Imaris 9.9.0 software. The estimated spot size was set to ≤0.7 µm to avoid recognition of more than one spot per vesicle. A threshold for spot recognition was set to avoid tracking of background signals. Autoregressive motion was used for tracking and a “maxDistance” was set to 1 µm with a “MaxGapSize” of 3 frames between each track. Tracks created by Imaris were occasionally fragmented and/or falsely connected to each other, therefore the “Edit Tracks” function was used to correct tracking of each spot. Imaris’ “statistics” function was used to extract data for number of tracks per cell, average track speed, and average track length.

### Statistical Analysis

Data were presented either as mean ± SEM or violin plots with median, first, and second quartile. For multiple comparisons, one‐way analysis of variance (ANOVA) with Tukey's *t*‐test was applied. For all panels shown, at least three independent experiments were performed. Representative experiments are shown. Data for relative fluorescence intensity were measured and extracted with the “profile” function of Zen Blue. GraphPad Prism 9.3 was used for all statistical analysis and *p* values less than 0.05 were considered to indicate significant differences (^*^ *p* < 0.05; ^**^ *p* < 0.01; ^***^ *p* < 0.001; ^****^ *p* < 0.0001); *p* > 0.05, non‐significant (NS).

## Conflict of Interest

The authors declare no conflict of interest.

## Supporting information

Supporting Information

Supplemental Video 1

Supporting Information

Supplemental Video 2

Supporting Information

Supplemental Video 3

Supporting Information

## Data Availability

The data that support the findings of this study are available from the corresponding author upon reasonable request.
