# Supplementary material for: Vesicle‐Associated Actin Assembly by Formins Promotes TGFβ‐Induced ANGPTL4 Trafficking, Secretion and Cell Invasion
Source: Adv Sci (Weinh). 2023 Jan 24;10(9):2204896. doi: 10.1002/advs.202204896 (PMC10037683; doi:10.1002/advs.202204896)
Supplement: Supplementary file 1 — Supporting Information [file ADVS-10-2204896-s002.pdf]

ADVANCED SCIENCE
Open Access

Supporting Information

for *Adv. Sci.*, DOI 10.1002/advs.202204896

Vesicle-Associated Actin Assembly by Formins Promotes TGFβ-Induced ANGPTL4 Trafficking, Secretion and Cell Invasion

Dennis Frank, Christel Jessica Moussi, Svenja Ulferts, Lina Lorenzen, Carsten Schwan and Robert Grosse**

Supporting Information

Vesicle-associated actin assembly by formins promotes TGFβ-induced ANGPTL4 trafficking, secretion and cell invasion

Dennis Frank[1,*], Jessica Christel Moussie[1,2,*], Svenja Ulferts[1], Lina Lorenzen[1], Carsten Schwan[1,#] and Robert Grosse[1,3,#]

Supplementary Figures and Videos:

Content:

Figure S1

Figure S2

Figure S3

Figure S4

Figure S5

Video S1

Video S2

Video S3

Figure S1

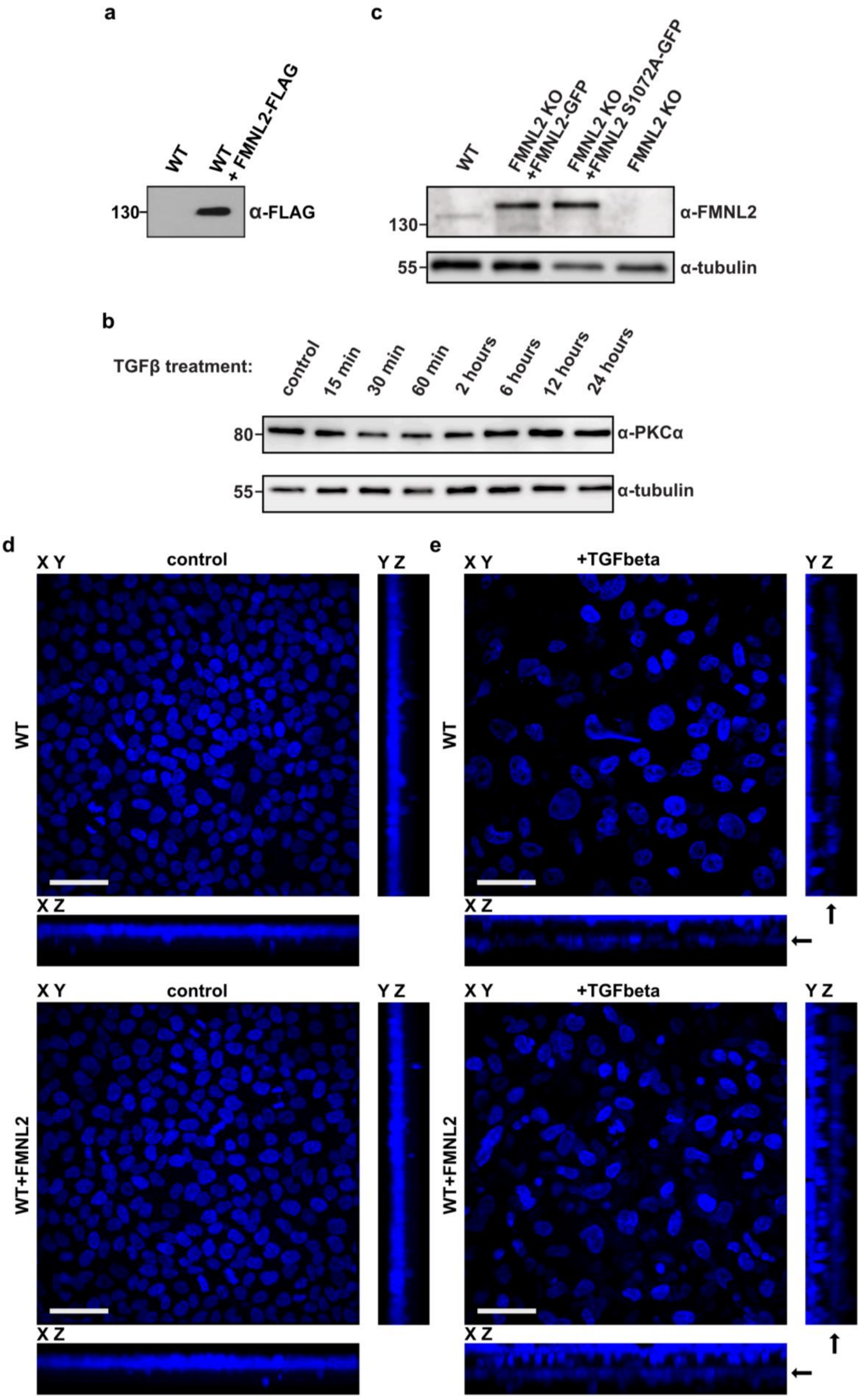
a
WT
WT + FMNL2-FLAG
130
α-FLAG
c
WT
FMNL2 KO +FMNL2-GFP
FMNL2 KO +FMNL2 S1072A-GFP
FMNL2 KO
130
α-FMNL2
55
α-tubulin
b
TGFβ treatment:
control
15 min
30 min
60 min
2 hours
6 hours
12 hours
24 hours
80
α-PKCα
55
α-tubulin
d
e
X Y
control
Y Z
+TGFbeta
WT
X Z
WT+FMNL2

Figure S1: a) Stable expression of FMNL2-FLAG in MCF10A cells. Western Blot analysis of FMNL2-FLAG expression in MCF10A WT cells with an anti-FLAG antibody. b) Western Blot analysis of PKCα expression in MCF10A wildtype cells after increasing durations of TGFβ treatments (4 ng/ml). c) Western Blot analysis of FMNL2 expression in MCF10A wildtype, FMNL2 KO and FMNL2 KO cells stably re-expressing FMNL2-GFP or FMNL2S1072A-GFP with an anti-FMNL2 antibody. d) MCF10A wildtype (upper panel) and FMNL2 overexpressing cells (lower panel) show no invasion into Matrigel under basal condition. e) TGFβ stimulation for 48 hours induced invasive behavior of MCF10A wildtype and FMNL2 overexpressing cells. Black arrows indicate invasion front. Quantification of invasion capacity shown in Figure 1, b. Scale bar = 50 µm.

Figure S2

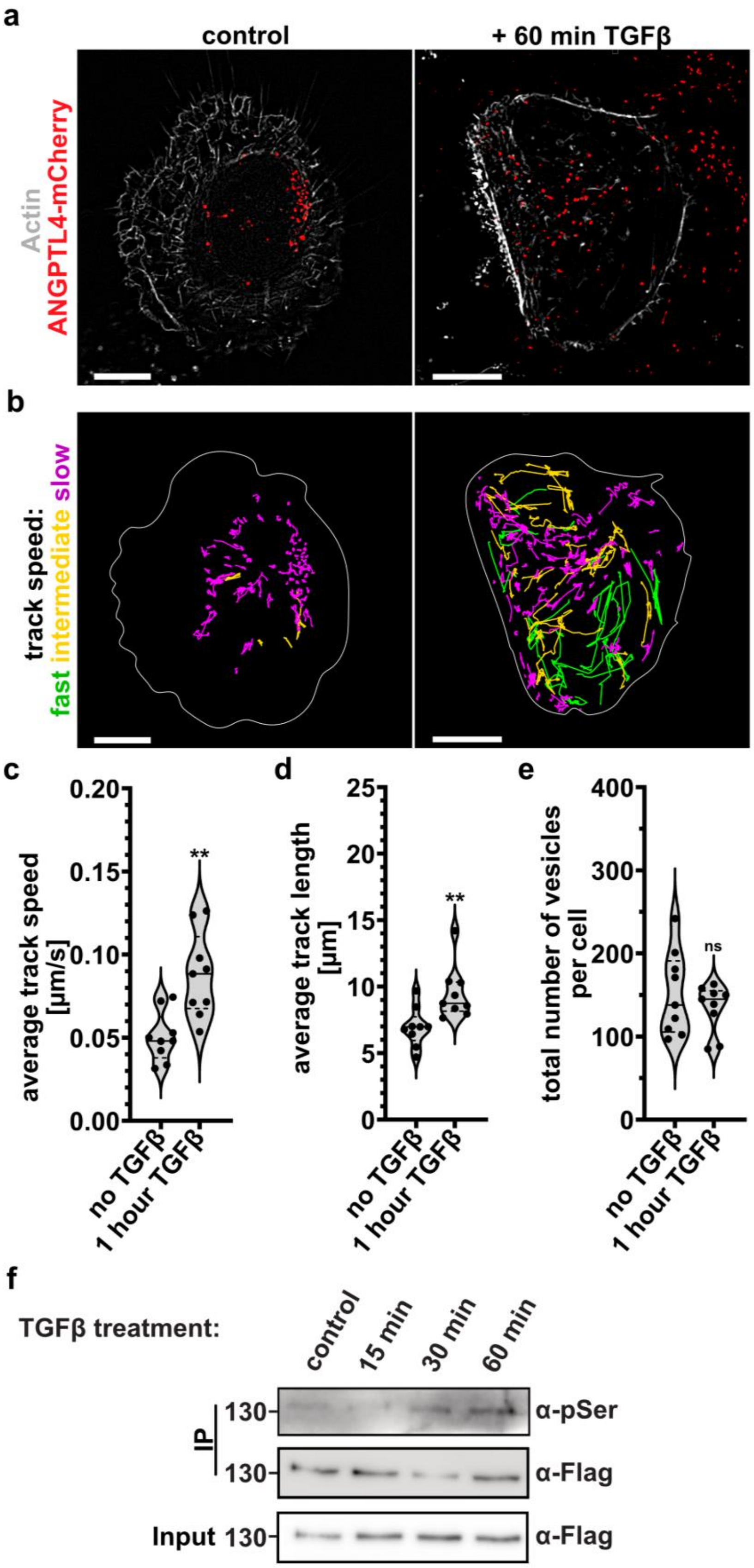
a
control
+ 60 min TGFβ
Actin ANGPTL4-mCherry
b
track speed: fast intermediate slow
c
average track speed [µm/s]
0.20
0.15
0.10
0.05
0.00
**
no TGFβ
1 hour TGFβ
d
average track length [µm]
25
20
15
10
5
0
**
no TGFβ
1 hour TGFβ
e
total number of vesicles per cell
400
300
200
100
0
ns
no TGFβ
1 hour TGFβ
f
TGFβ treatment:
control
15 min
30 min
60 min
IP
130
α-pSer
130
α-Flag
Input 130
α-Flag

Figure S2: Short-term TGFβ treatment induces ANGPTL4 trafficking. a) MCF10A wildtype cells were transiently transfected with ANGPTL4-mCherry and Actin-Chromobody SNAP. Cells were subjected to super-resolution live cell imaging with enhanced temporal resolution (burst mode, sliding processing) in absence of TGFβ (left panel) or after one-hour TGFβ stimulation (right panel). Scale bar = 10 µm. b) In the resulting sequential images ANGPTL4-positive vesicles were tracked. The trajectories of ANGPTL4-mCherry vesicles tracked for at least 60 seconds are shown. Trajectories were categorized by their average track speed into three groups: magenta = slow speed (0 - 0.10 µm/s), yellow = intermediate speed (0.10 - < 0.15 µm/s), green = fast speed (≥ 0.15 µm/s). Grey line indicates cell border based on the actin cytoskeleton. Scale Bar = 10 µm. c, d, e) In cells treated as in (a), the average track speed (c), average track length (d) and total number of vesicles per cell (e) was quantified. (n = 9). Data are shown as violin plots with median (solid line), first and second quartile (dashed lines), spots represent individual values. One-way ANOVA with Tukey's multiple comparisons test was used for statistical analysis (** =p<0.01). f) Immunoprecipitation of FMNL2-FLAG with an anti-FLAG antibody of stably FMNL2-FLAG-expressing MCF10A cells after short-term stimulation with TGFβ. Phosphorylated FMNL2 was detected by western blot with a phospho-(Ser)-specific antibody.

Figure S3

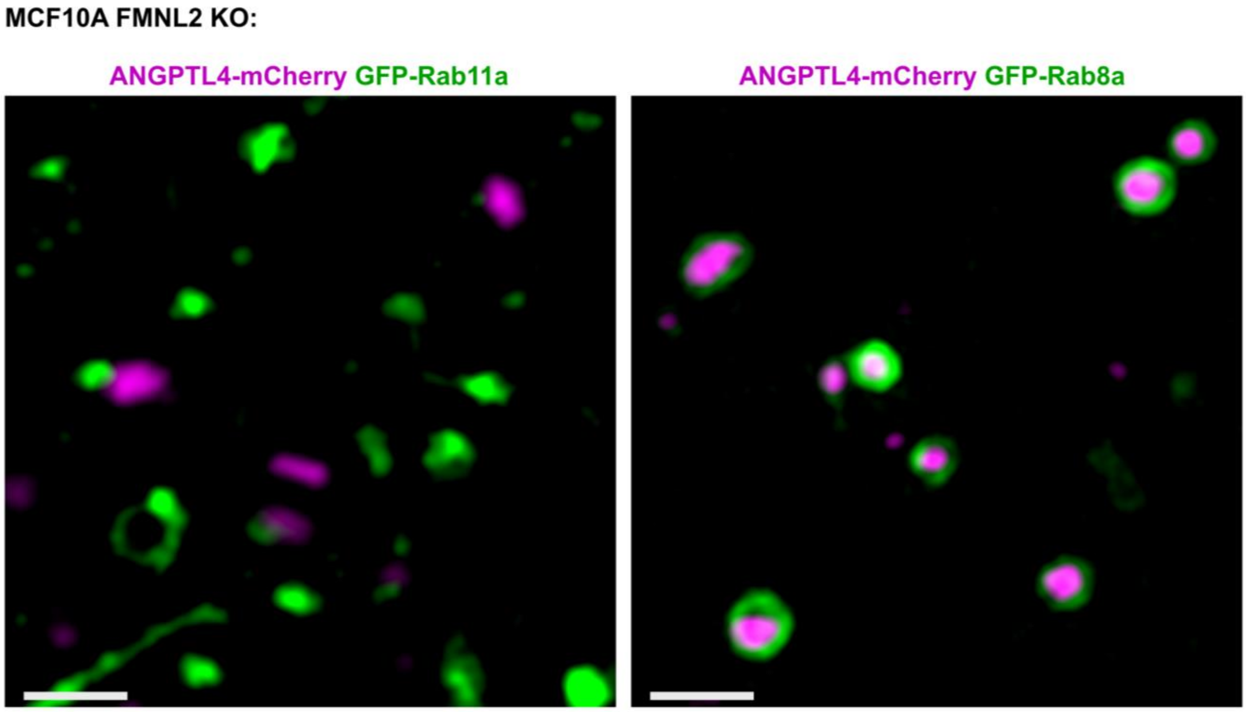

Figure S3: GFP-Rab8a colocalizes with static ANGPTL4-mCherry vesicles in MCF10A FMNL2-KO cells. Sections of MCF10A FMNL2-KO cells transfected with ANGPTL4-mCherry and either GFP-Rab11a (left panel) or GFP-Rab8a (right panel). SIM images show colocalization of ANGPTL4-mCherry with GFP-Rab8a, but not with GFP-Rab11a. Scale bar = 1 µm.

Figure S4

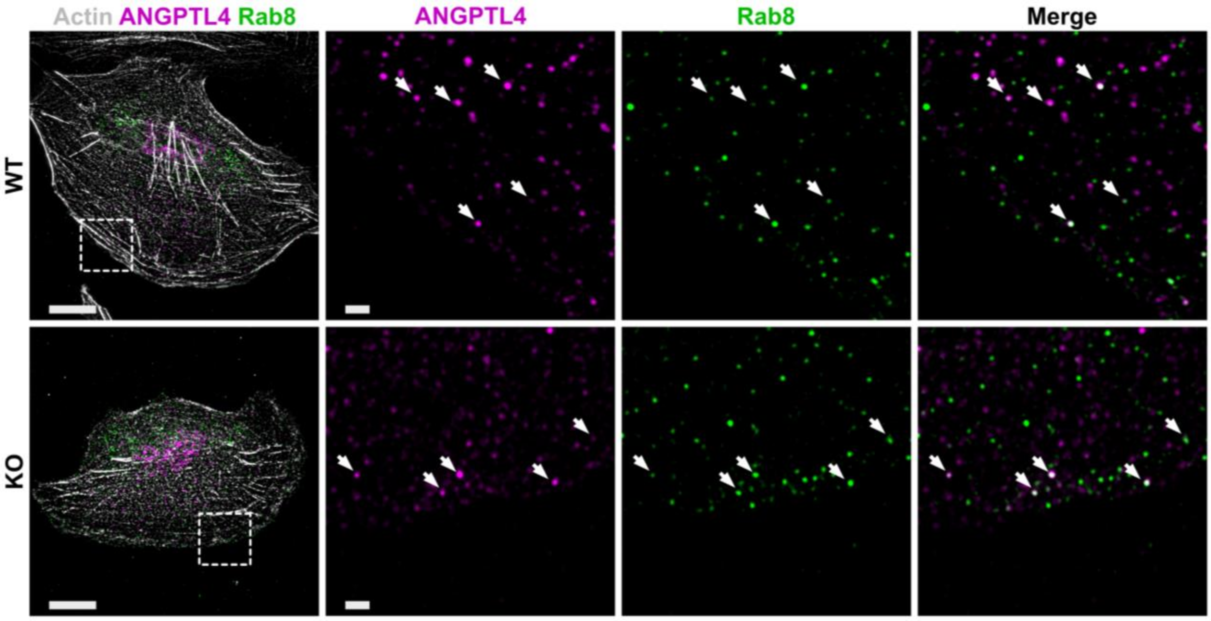

Figure S4: Endogenous ANGPTL4 colocalizes with Rab8. Immunostaining of ANGPTL4 (magenta) and Rab8 (green) in WT (MCF10A wildtype) and MCF10A FMNL2-KO cells that were treated with TGFβ for 24 h. Phalloidin was used to visualize F-actin (white). White box indicates magnified region. White arrows indicate colocalizing ANGPTL4 vesicles with Rab8. Scale bars: overview = 10 µm, magnifications = 1 µm.

Figure S5

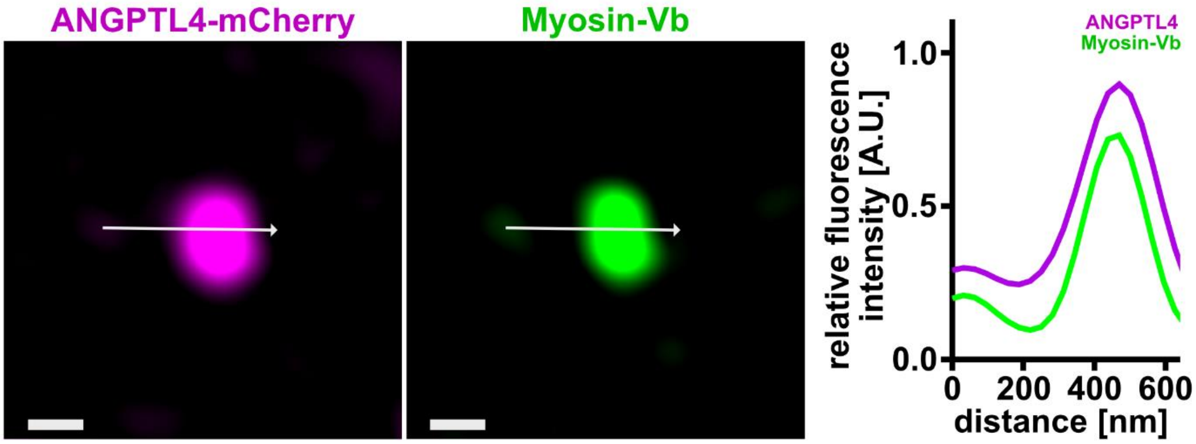

Figure S5: ANGPTL4 colocalizes with Myosin-Vb in MCF10A FMNL2-KO cells. Immunostaining of Myosin-Vb (green) in MCF10A FMNL2-KO cells that were treated with TGFβ for 24 hours after transfection with ANGPTL4-mCherry (magenta). White arrows indicate line scan direction and length. Line-scan profile shows normalized fluorescence intensity of ANGPTL4 (magenta) and Myosin-Vb (green). Scale bar = 200 nm.

Video S1

ANGPTL4 vesicle trafficking in MCF10A wildtype cells. Super-resolution live cell imaging after treatment with TGFβ for 24 hours and transfection with ANGPTL4-mCherry (red) and actin-chromobody SNAP (white). Time = m:ss. Scale bar = 10µm.

Video S2

Impaired ANGPTL4 trafficking in MCF10A FMNL2 KO cells. Super-resolution live cell imaging after treatment with TGFβ for 24 hours and transfection with ANGPTL4-mCherry (red) and actin-chromobody SNAP (white). Time = m:ss, Scale bar = 10µm.

Video S3

Super-resolution live cell imaging of FMNL2 (green) polymerizing actin (white) at an ANGPTL4-containing vesicle (magenta) in MCF10A KO (+FMNL2-GFP) cells. Time = m:ss. Scale bar = 200nm.
