# Supplementary figures and images for: Vesicle‐Associated Actin Assembly by Formins Promotes TGFβ‐Induced ANGPTL4 Trafficking, Secretion and Cell Invasion
Source: Adv Sci (Weinh). 2023 Jan 24;10(9):2204896. doi: 10.1002/advs.202204896 (PMC10037683; doi:10.1002/advs.202204896)

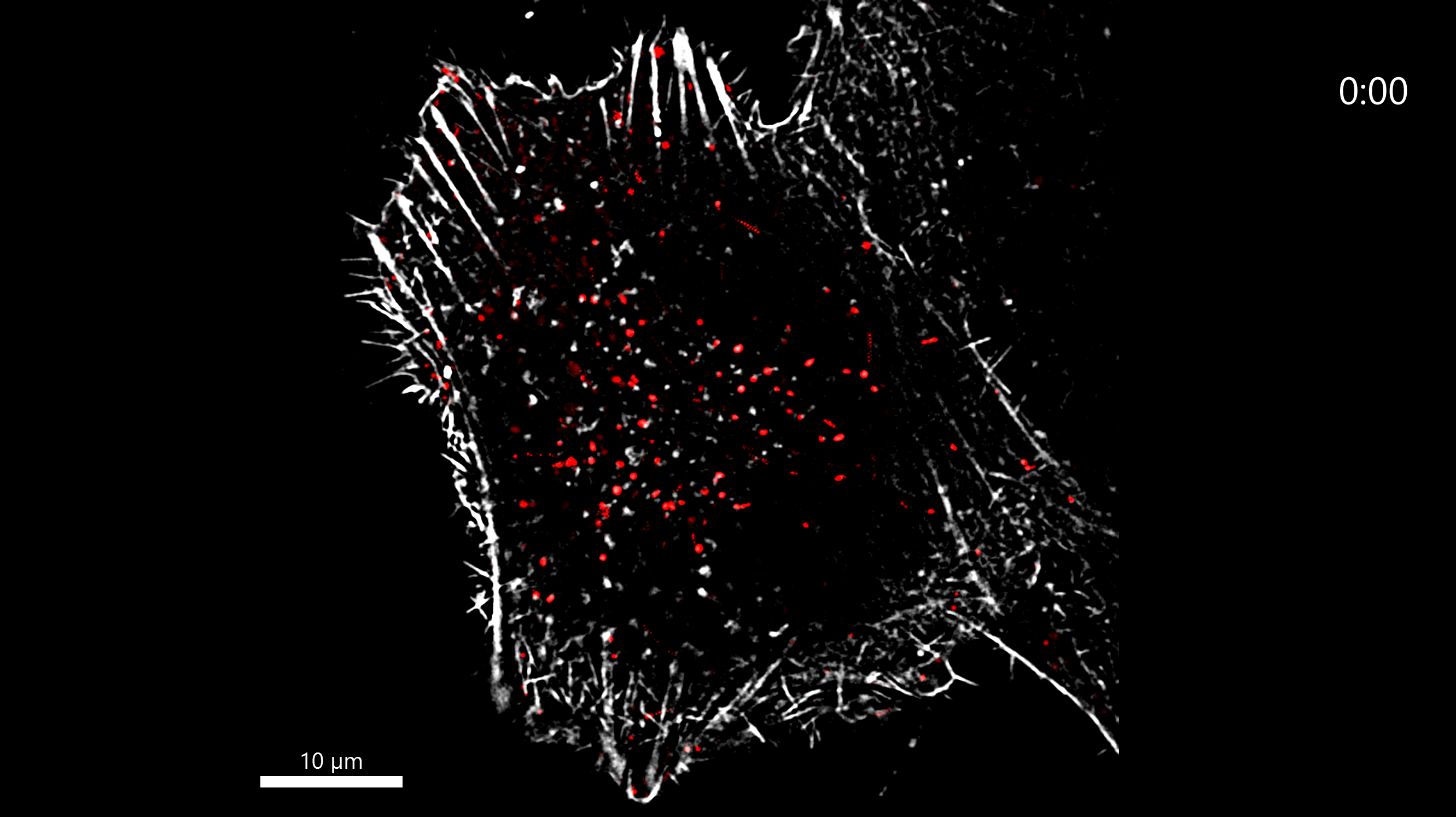

Supplement: Supplementary file 3 — Supporting Information [file ADVS-10-2204896-s006.tif]

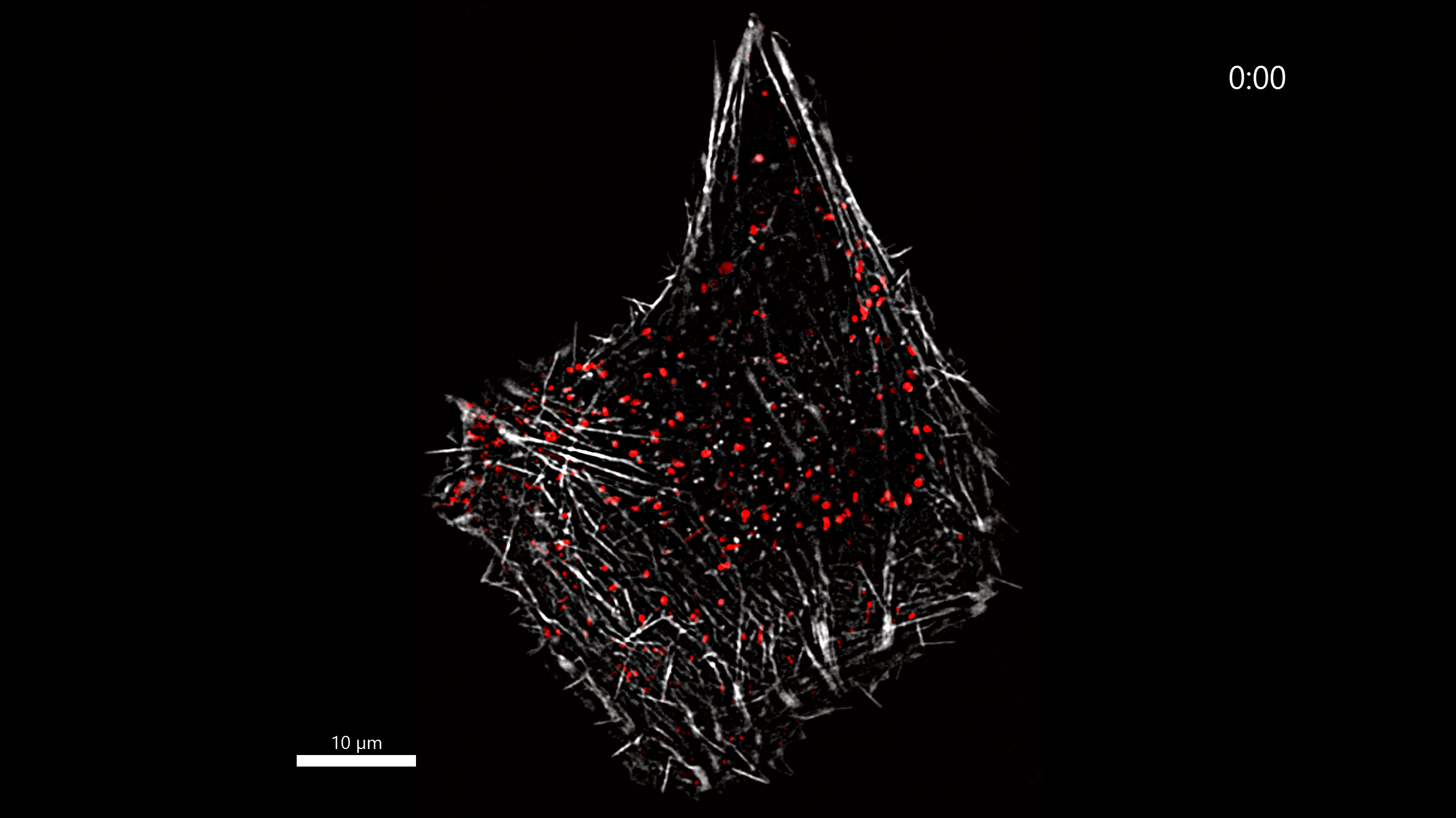

Supplement: Supplementary file 5 — Supporting Information [file ADVS-10-2204896-s004.tif]

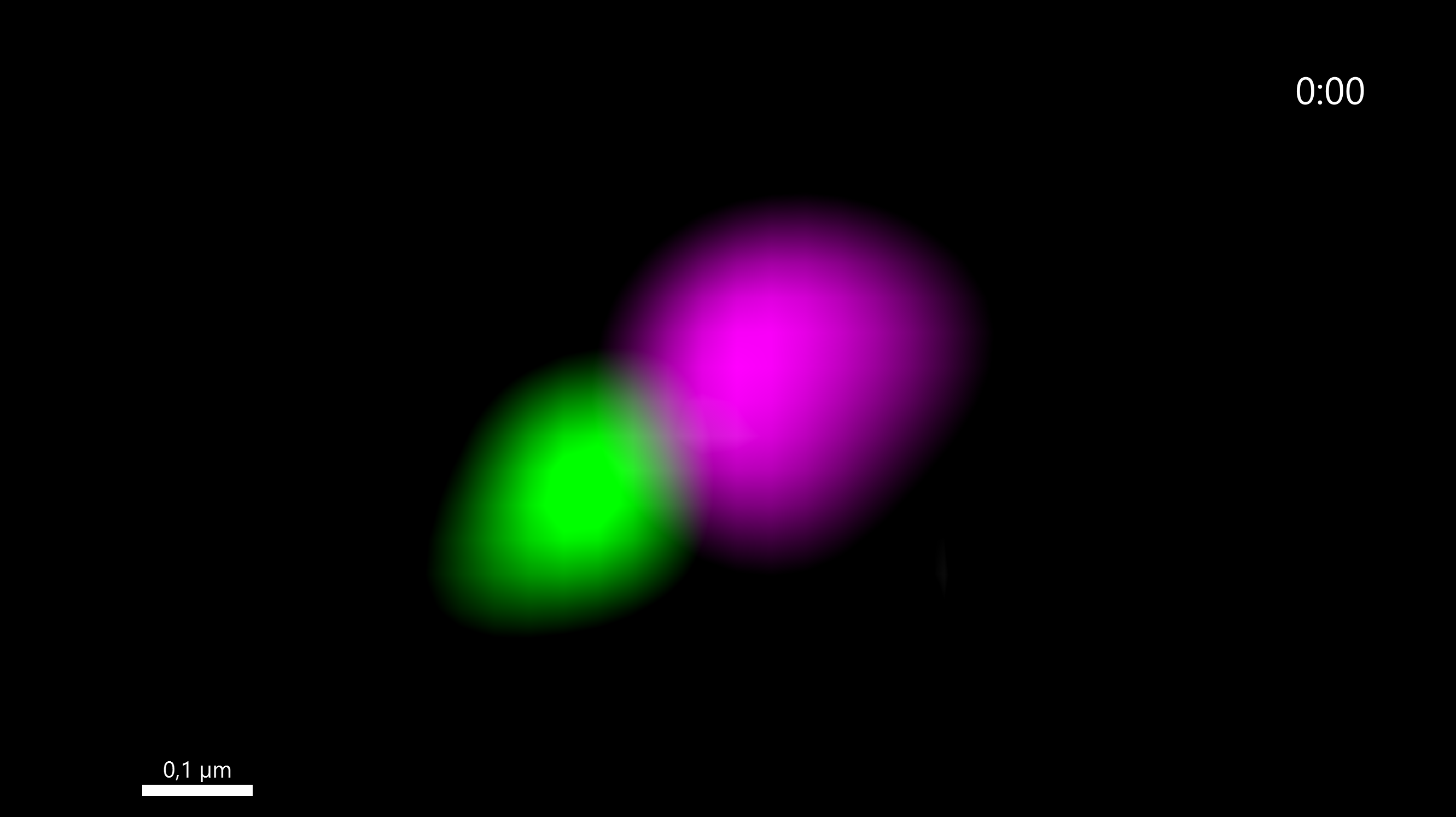

Supplement: Supplementary file 7 — Supporting Information [file ADVS-10-2204896-s005.tif]
